# Indoor Path-Planning Algorithm for UAV-Based Contact Inspection

**DOI:** 10.3390/s21020642

**Published:** 2021-01-18

**Authors:** Luis Miguel González de Santos, Ernesto Frías Nores, Joaquín Martínez Sánchez, Higinio González Jorge

**Affiliations:** 1 CINTECX, GeoTECH Group, Campus Universitario de Vigo, University of Vigo, As Lagoas, Marcosende, 36310 Vigo, Spain; efrias@uvigo.es; 2 GeoTECH Group, Department Natural Resources and Environmental Engineering, Campus Lagoas, School of Aerospace Engineering, University of Vigo, 32004 Ourense, Spain; higiniog@uvigo.es

**Keywords:** autonomous navigation, contact inspection, NDT, UAV, payload, industrial inspection

## Abstract

Nowadays, unmanned aerial vehicles (UAVs) are extensively used for multiple purposes, such as infrastructure inspections or surveillance. This paper presents a real-time path planning algorithm in indoor environments designed to perform contact inspection tasks using UAVs. The only input used by this algorithm is the point cloud of the building where the UAV is going to navigate. The algorithm is divided into two main parts. The first one is the pre-processing algorithm that processes the point cloud, segmenting it into rooms and discretizing each room. The second part is the path planning algorithm that has to be executed in real time. In this way, all the computational load is in the first step, which is pre-processed, making the path calculation algorithm faster. The method has been tested in different buildings, measuring the execution time for different paths calculations. As can be seen in the results section, the developed algorithm is able to calculate a new path in 8–9 milliseconds. The developed algorithm fulfils the execution time restrictions, and it has proven to be reliable for route calculation.

## 1. Introduction

Unmanned aerial vehicles (UAVs) are gaining more and more significance due to their adoption in a wide variety of engineering fields, such as surveying, monitoring, or precision agriculture [1]. UAVs have proven to be a key instrument for large-scale infrastructure maintenance, especially for bridge inspection where they have been applied to detect corrosion and cracks [2] or to monitor the condition state of structure joints [3], among others. In most cases, these inspections are carried out with remote sensing payloads, including RGB cameras [4], LiDAR (Light Detection And Ranging) sensors [5], or thermographic cameras [6]. 

According to the literature, UAVs along with camera sensors, smartphones, and mobile systems arise as a modern next-generation technology for SHM (structural health monitoring) as opposed to traditional methods based on contact sensors that involve time- and labor-intensive installation and maintenance [7]. In order to solve these drawbacks and to combine the strengths of both technologies, UAV-based contact inspection systems have been proposed [8,9]. A challenging issue for UAVs to implement contact inspection comes with the lack of robustness in GNSS (Global Navigation Satellite System) based location, such as GPS, in the vicinity of a large structure [10]. Therefore, smart payloads providing UAVs with location (position and orientation) and collision mitigation are proposed as a mechanism to convert general-purpose drones into contact inspection systems [11].

In addition to that, location in GPS-denied environments supports indoor positioning and navigation, an emerging field with a wide variety of applications for private and public areas and from casual personal use to critical emergency response [12]. Although some systems are based on the availability of an infrastructure based on IoT sensors [12] or camera-based tracking systems [13], a more general solution requires systems to calculate the indoor map based on their payload sensors, such as LiDAR [14], deep learning-aided monocular cameras [15], optical and mass flow sensors [16], or multi-sensor fusion [17].

Obtaining the map is the basis for decision making about the optimal mission path to be followed by the UAV between source and destination, the so-called path planning procedure [18]. In 1959, Dijkstra [19] presented a graph searching algorithm that incorporates heuristic information to the mathematical model. This work defines the A* path planning algorithm, which is the basis of many of the path-planning algorithms developed nowadays. The aim of path planning consists of obtaining the mission graph and the methods, which are classified into five categories [20]: Sampling-based, node-based, mathematical model, bio-inspired, and multi-fusion-based algorithms. Among them, sampling-based algorithms use a priory 3D information of the environment, dividing the space into a structured set of cells, which supports the sequential computation of the path between adjacent cells and the obstacle avoidance [18]. The autonomous navigation of UAVs in the context of GNSS-denied zones is based on local information. Such local information may be obtained from an RGB-D Kinect camera to generate an analytical path computation based on guaranteeing a safe navigation with a radial buffering function around obstacle candidates [21]. Valenti et al. [22] presents a computer vision system based on the exploitation of visual information about 3D surroundings of the UAV that is the basis to find a precise localization and the shortest path to reach the goal using a simultaneous localization and mapping (SLAM) fashion. Computer vision is a well-established technique that supports the use of cooperative UAVs and UGVs (unmanned ground vehicles) to obtain complementary points of view of the scene for path discovery [23]. Other algorithms use the BIM (building information modeling) models [24] to create the graph and then calculate the paths.

Several 2D path planning algorithms have been developed for UGV systems that use different approaches, such as resistive-grids based path-planning algorithms, Q-learning algorithms, rapidly exploring random tree algorithms, Dijkstra, and A* path planning algorithms [25,26]. Huang et al. [27] introduced a path planning algorithm for multi-robots in a structured hospital environment with two different 2D path planning algorithms, one for the corridors where a graph search algorithm is used, and another for the rooms where an artificial potential field method is used. Maoudj et al. [28] proposed an EQL (efficient Q-learning) path planning algorithm that is able to overcome the QL limitations to ensure an optimal collision-free path. Hernández et al. [29] created a Resistive Grid that transforms the navigable space into an electric circuit where each component of the grid behaves as a resistance and its value depends on its position in the grid regarding the target position. Ambroziak et al. [30] presented a multicriterial minimization method to calculate optimal paths. Underground mine exploration is an important application of path planning algorithms. In recent years, several contributions to this topic have been introduced. Dang et al. [31] introduced a two-level navigation system, the first one for local exploration (near to the vehicle) and the second one for the global planner. Li et al. [32] presented a probabilistic navigation algorithm for exploration of indoor environment, focused on this case of underground mine exploration. It implements a navigation system based on graph navigation.

This work presents a path planning algorithm developed to enable a UAV to navigate in an indoor environment to perform contact inspection tasks. The objective of this work is to develop an algorithm for path planning to be executed in real time, meaning that the system is able to calculate new paths in a few milliseconds. The only input for the algorithm is a point cloud obtained with Zeb-Revo LiDAR [33]. In order to calculate the path between two points as fast as possible, the method has been divided into two main parts, one of them with almost all the computational load that is pre-processed, obtaining new data that are used to calculate new paths quickly by the second part. In addition, the algorithm segments the entire point cloud into rooms, analyzing each one separately. In this way, when a new path is calculated, just the info from the rooms that the system has to go through is used, making the method more efficient. Preliminary results have been published in a congress [34]. During the development of this previous path planning algorithm, authors noticed that the algorithm was too slow to be implemented in a real study case, as it does not fulfil the execution time restrictions to be considered as a real-time path planner. This previous work focused on the navigation map generation, without segmenting the point cloud into rooms and without the division of the algorithm in a pre-processing step and a real-time path calculation. This new work is an improvement of the previous work, making it more efficient and presenting a real-time path planning algorithm. The importance of executing the path planning algorithm in real time is that the vehicle navigates in an indoor environment with mobile elements or even shared spaces with humans. For the system optimization, the vehicle has to be able to calculate new routes quickly in order to interact with a dynamic environment, such as industrial buildings, aircraft hangars, hospitals, or similar. The presented path planning algorithm is a modification of the A* algorithm, adapting it to work in a 3D environment, as it was developed to be used with UAVs. The main novelties of this work are the room segmentation, the navigation graph generated to navigate from one room to another, and the adaptation of the A* algorithm to make it work in real time, dividing the method into two parts, a pre-processing step with almost all the computational load and a path planning step that is able to calculate new paths in real time. In addition, this path planning has been developed to perform contact inspection tasks with a UAV, which is a novel implementation of this vehicle.

The developed algorithm is 3D path planning, meaning that the algorithm has been designed to calculate paths for a UAV to navigate in a 3D environment. It can also be modified to be implemented to navigate in a 2D environment, using the 3D information given by the point cloud. In this way, it can be adapted to be used by other kinds of ROVs (remotely operated vehicles), like a UGV (unmanned ground vehicle) or other autonomous surface vehicles. 

This manuscript is organized as follows: Section 2 introduces the methodology and explains the pre-processing and the path planning steps. Section 3 reports the results obtained with the developed algorithm, measuring its processing time. Section 4 shows the conclusions of the work and future works.

## 2. Methodology

**Figure 1:**
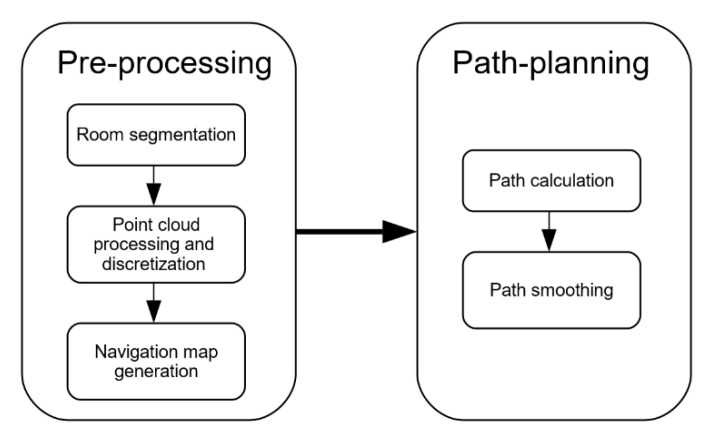
General workflow of the method**.**

As aforementioned, the methodology has been divided in two main parts (Figure 1). First, a pre-processing step adapts the point cloud to calculate the indoor navigable space map that supports the successive path-planning calculation. This task breakdown results in a real-time calculation of new routes by moving the computational efforts to the pre-processing step, which is executed only once.

The pre-processing part was divided in three main steps: Room segmentation, point cloud processing and discretization, and navigation map generation. In room segmentation, the method used to locate the doors of the point cloud and segment it into rooms is explained. The point cloud processing section explains the methods used to align the point cloud, segment the floor and ceiling, and the discretization method applied. Once the point cloud is segmented and discretized, the navigation map is generated. With this map, the path-planning algorithm is able to calculate, in real time, new paths. These paths can be smoothed, making them more efficient for the UAV navigation. 

### 2.1. Point Cloud Pre-Processing

This first part of the method deals with the processing of the spatial information contained in a point cloud to obtain a 3D navigation map, which is to be used for the path planning. As shown in Figure 1, the pre-processing is divided into three main tasks: Room segmentation, point cloud processing and discretization, and navigation map generation for each room. 

#### 2.1.1. Room Segmentation

The method starts with room segmentation. This step consists of splitting an indoor point cloud into segments corresponding to rooms. A room is defined as an indoor space enclosed by structural elements and connected to other spaces by doors. The purpose of room segmentation is to create a general navigation graph, considering rooms and their connections, which will be used as the starting point for the graph generation for UAV navigation. 

**Figure 2:**
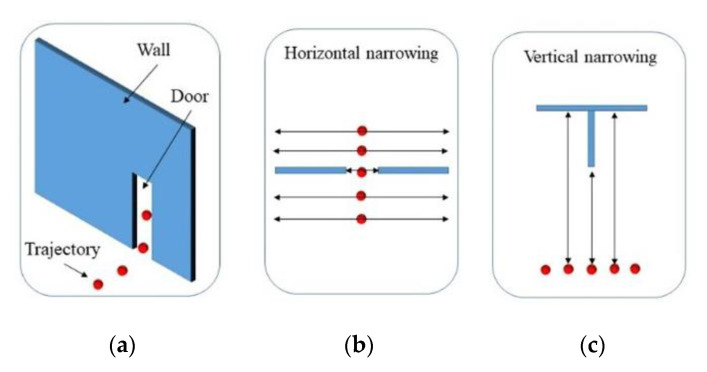
Room segmentation scheme. Red points: Trajectory positions. Blue components: Structural elements. (**a**) General scheme. (**b**) Horizontal spatial narrowing because a door is crossed. (**c**) Vertical spatial narrowing between the trajectory positions and structural components such as ceiling or walls.

The implemented room segmentation extends the previous work proposed by Díaz-Vilariño et al. [35]. As in the previous approach, the input data consist of a point cloud acquired with an indoor Mobile Mapping System (iMMS) and the trajectory followed during the surveying, both related by their timestamps. Besides, room segmentation is based on door detection. A door is assumed as a narrowing in the space connecting two rooms. Unlike the previous approach, in this work, doors are detected from a double-check process consisting of finding vertical and horizontal narrowings along the trajectory (Figure 2). 

As it has been said in the Introduction section, the point clouds used in these works have been acquired with an indoor Mobile Laser Scanner system. This kind of laser scanner acquired points continuously, referencing them to a time stamp, and calculates the trajectory of the acquisition using an inertial sensor and the spatial data acquired, also referencing it to a time stamp. This trajectory is composed by several points, which can be filtered by time stamp, as can be seen in Figure 2. The trajectory height depends on the person carrying the laser scanner, which is basically a backpack. Each one of these trajectory positions is studied, generating a study area around them, defining it as a sphere centered in the trajectory area with a radius of 1 m. The radius of the sphere is used to filtrate the points of the point cloud that are going to be used to locate narrowings, and just the points inside the sphere are used. A large radius will make the algorithm slow, and a short radius may cause some narrowings to be missed outside the filter sphere.

For the horizontal spatial narrowings study, Figure 2b, a new study area is defined using the previous filtrated data. This area is defined by the horizontal plane that contains the trajectory point. This plane is shifted along the z axis in the positive and negative distance; in this case, a shift of ±0.5 m is used. Then, the points inside the study area are clustered using a DBSCAN algorithm. If two clusters are collinear with a trajectory position, the distance between clusters is assigned to the trajectory position as a measure of width. The maximum width of door $w_{max}$ is set-up to avoid over-detection along the corridors. If the width measured is lower than the maximum door width $w_{max}$, the studied trajectory position is set as a possible door position that is studied again looking for vertical narrowings.

**Figure 3:**
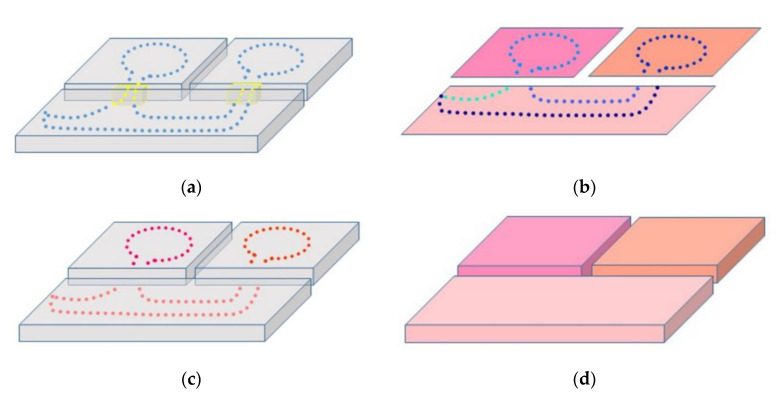
Room segmentation process. (**a**) Raw point cloud (grey) and trajectory classified as door (yellow) and nondoor positions (blue). (**b**) Clustered room regions and projected trajectory segments. (**c**) Labeled trajectory and unlabeled point cloud (grey). (**d**) Segmented point cloud from labeled trajectory positions.

For the vertical spatial narrowings study, Figure 2c, just the trajectory positions selected as possible doors in the previous step are studied. In this vertical study, the system looks for narrowings caused by the wall section located above the door. This analysis estimates the ceiling height for each trajectory position studied. To make this analysis, just the points above the horizontal plane at the height of the trajectory positions are used. This height is calculated using the z-histogram, locating the maximum of the histogram. The points inside the study area are clustered using a DBSCAN algorithm, as in the previous case. If the possible door position has a cluster above the trajectory position with a height lower than the ceiling height calculated, the trajectory position is marked as a door position. More than one consecutive trajectory position can be selected as a door position, referring to the same door. In order to avoid this problem, the trajectory positions are filtered, selecting just one trajectory position as the door position. Due to how the acquisition of the point cloud is carried out, the LS (laser scanner) can go through the same door twice, but the filtering described previously only filters the doors found in consecutive positions of the trajectory. Algorithm 1 resumes the door location process.
**Algorithm 1** Door Location Algorithm**Require:** Point Cloud: PC, Trajectory positions: TnT = length(T);**for** i = 1:nT      Points = SelectTimeStamp(PC, T(i));      studyPoints = SelectSphere(rSphere, Points);      //*Horizontal spatial narrowings study*      PointsHN = SelectVerticalShifted(verticalOffset, studyPoints);      cluster = DBSCAN(PointsHN);      **if** (LocateHorizontalNarrowings(cluster) == true) **then**            //*Vertical spatial narrowings study*            PointsVN = SelectAbovePlane(height(T(i), studyPoints);            ceilingHeight = Max.HistogramZ(PointsVN);            cluster = DBSCAN(PointsVN);            **if** (LocateVerticalNarrowings(cluster, ceilingHeight) == true) **then**                  Doors = AddDoor(i);            **endif**      **endif****endfor**Doors = ConsecutiveDoorsFiltration(Doors);

Once doors are identified on trajectory, the trajectory is partitioned into ‘segments’ by removing the positions corresponding to doors (Figure 3b). As the connectivity between adjacent rooms is provided by doors, one segment can only correspond to a room and two consecutive segments cannot belong to the same room. In addition, a room can be acquired from several trajectory segments as it is shown in the largest room of Figure 3b. Trajectory labeling is performed by projecting its positions on the room regions obtained from a three-step process. First, points composing the floor are extracted from the point cloud. In the second step, a circular buffer centered on door position is created for every door. Floor points falling into buffers are removed to break connectivity between rooms at the floor level. In the last step, remaining floor points are clustered using the DBSCAN algorithm so that each cluster of floor points belongs to a room region. Then, trajectory segments are labeled with a room attribute based on the semantics of the floor segment they are projected onto (Figure 3c). 

Finally, the timestamps of the labeled trajectory are used to segment the point cloud into rooms (Figure 3d). This approach is simpler than applying geometry-based segmentation, including those based on extracting structural elements such as walls and doors. 

**Figure 4:**
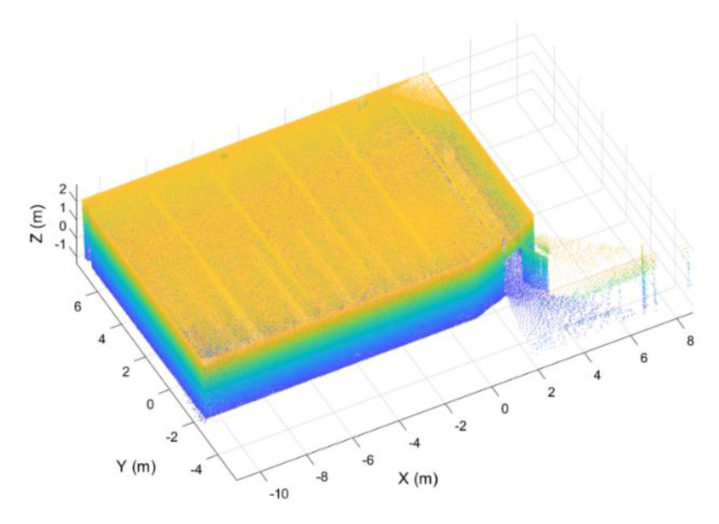
Room segmented.

Due to this segmentation method, outdoor points collected from an indoor location are mislabeled due to their timestamp and trajectory origin (Figure 4). A straightforward filter has been developed to solve this mislabeling issue.

**Figure 5:**
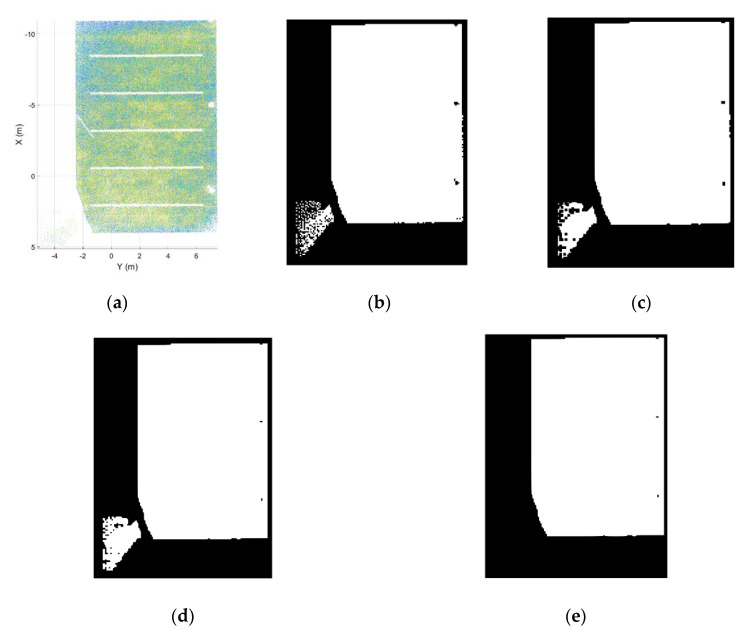
Ceiling delimitation for room segmentation. (**a**) Segmented point cloud of the ceiling. (**b**) Binary image of the ceiling point cloud. (**c**) Binary image after a morphological closing. (**d**) Binary image after a morphological dilatation. (**e**) Binary image of a room delimitation.

The filter exploits the shadows on the ceilings caused by the surroundings of the wall where the door is fit. First, the ceiling of the room is segmented using the Z-histogram of the point cloud and a RANSAC (Random Sample Consensus) algorithm [36]. This ceiling plane is projected onto the XY plane and transformed to a binary image using a 2D uniform grid with a predefined cell size, 10 cm for this study. The grid is populated with a point density gate, obtaining a “1” value when the number of points in the cell is above $nMin_{points}$ and a “0” value otherwise (Figure 5). The resulting image is afterward filtered with a morphological closing using a structuring element object consisting of a 4-pixel square. The objective of this filter is to label small areas that do not contain points but belong to the room. To ensure that all the room points are correctly labeled, a morphological dilation is applied, using the same structuring elements. In this way, the points that are close to the limits of the room are within the delimited area of the binary image. After this process, two separated areas are defined in the image, so the next step is to choose which of these two areas correspond to the room. This is done by selecting the element of the binary image with the largest area. 

**Figure 6:**
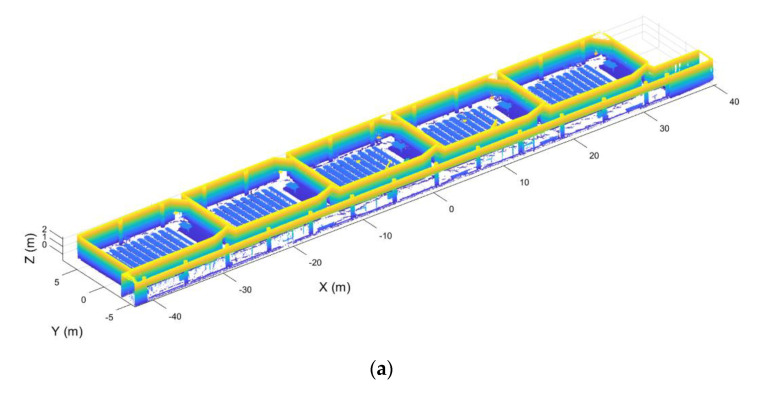

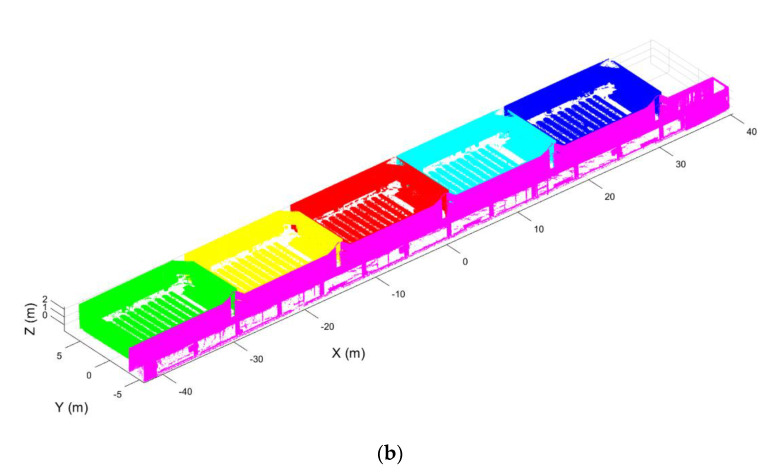
Point clouds without ceiling and floor segmented into rooms. (**a**) Initial point cloud, colored by height, before the segmentation. (**b**) Final point cloud labeled by rooms, each room with a different color.

After the delimitation of the room area (Figure 6), the 2D grid supports the classification of the room points.

#### 2.1.2. Point Cloud Pre-Processing 

In this section, the processing algorithm to be applied to the point cloud is explained. We consider as input the segmented point cloud where each point has been labeled with the room it belongs to, thus enabling us to obtain a point cloud per room.

**Figure 7:**
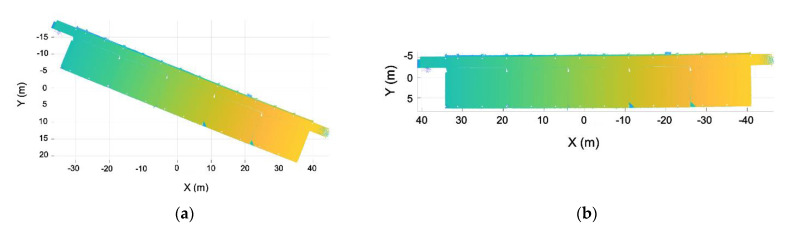
Point cloud alignment with the X and Y axes. (**a**) Point cloud before the alignment. (**b**) Point cloud after the alignment.

This step is based on the assumption that a building is typically composed of parallel and perpendicular walls, as shown Figure 6. With the aim of structuring the point cloud in voxels and to optimize such discretization, the walls of the building have been aligned with the X and Y axes. As a result, voxels are parallel to the walls, minimizing the number of voxels needed to discretize the entire point cloud. To achieve the alignment of the point cloud, the normal vector of the largest wall of one room is used, aligning this vector with the Y axis. This is done using the RANSAC algorithm to calculate the plane of the wall, using the same method developed in previous work [37]. With the normal vector of this plane $w=\left(nw_{x},\;nw_{y},\;nw_{z}\right)$, the angle formed by the normal vector and the Y axis is calculated using Equation (1). Once the angle is calculated, the homogeneous transformation matrix is defined (Equation (2)). This homogeneous transformation is applied to the point cloud of all the rooms (Figure 7).

(1) $$\alpha \;=atan\left(\frac{nw_{x}}{nw_{y}}\right)$$

(2) $$Rot\left(z,\alpha \right)=\;\left(\begin{array}{ccc} \cos\left(\alpha \right) & -\sin\left(\alpha \right) & 0\\ \sin\left(\alpha \right) & \cos\left(\alpha \right) & 0\\ 0 & 0 & 1 \end{array}\right)$$

**Figure 8:**
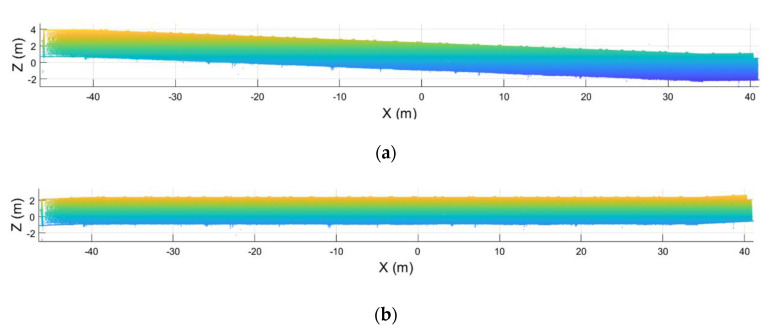
Point cloud leveled. (**a**) Point cloud before the leveling. (**b**) Point cloud after leveling.

A similar process is applied to the inclination of the point cloud. In this case, the plane of the ceiling is calculated, aligning its normal vector with the Z axis (Figure 8).

**Figure 9:**
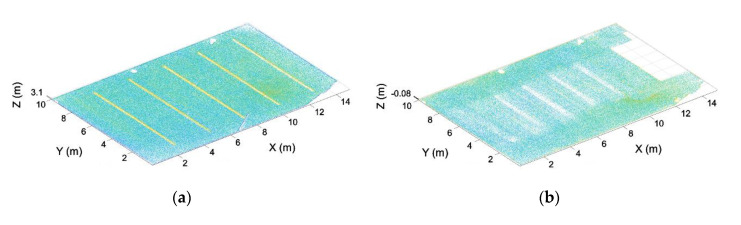

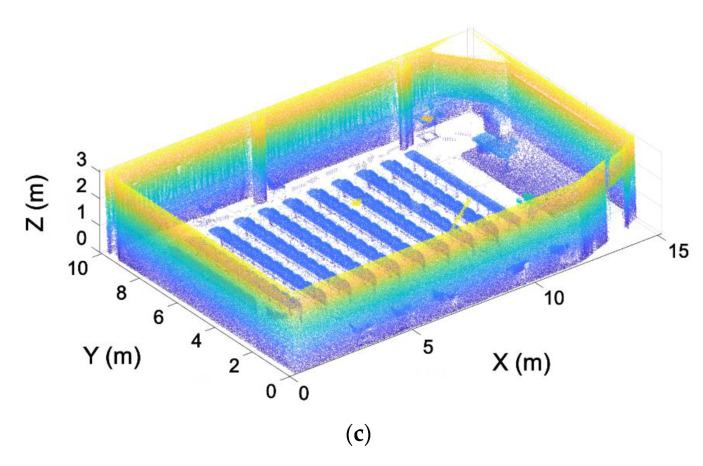
Point cloud segmentation into ceiling, floor, and room. (**a**) Ceiling point cloud. (**b**) Floor point cloud. (**c**) Room point cloud.

Once the point is segmented and aligned, each room point cloud is discretized in order to calculate a navigation map per room. The discretization process is similar to the one developed in a previous work [37]. The first step consists of segmenting the point cloud in three parts: Ceiling, floor, and room (Figure 9). First, and based on the Z-histogram, we calculate the height of the ceiling. Then, the planes for floor and ceiling are segmented using the RANSAC algorithm. Finally, the room point cloud is defined as the points that are located between these two planes, filtering all the points above the ceiling or below the floor. 

As aforementioned, the point cloud for each room is next structured through a voxelization, where data are divided in cubes of a determined size and labeled with the space occupancy. In this work, four different labels are used for voxel classification:

- Empty: Voxels that do not contain points inside.
- Occupied: Voxels that contain points inside.
- Security-offset: Empty voxels that are near an occupied one, and therefore, are not navigable by the drone.
- Exterior: Empty voxels that are outside the room.

Defining an appropriate voxel size is fundamental, because it implies a trade-off between the loss of detail in the 3D geometric information of the point cloud and the computational cost. The voxelization is stored in a matrix named Mv(t), that is, a 3-dimensional matrix where each cell represents a voxel. Once the voxelization matrix is created, voxels are classified in “Occupied” and “Empty” voxels based on whether the number of points is above a threshold or not, respectively. The next step deals with voxels outside the room, which are labeled as “Exterior” based on the 2D binary image created for point cloud segmentation and described in the previous section. This classification is applied only to “Empty” voxels; “Occupied” voxels are not re-classified. 

**Figure 10:**
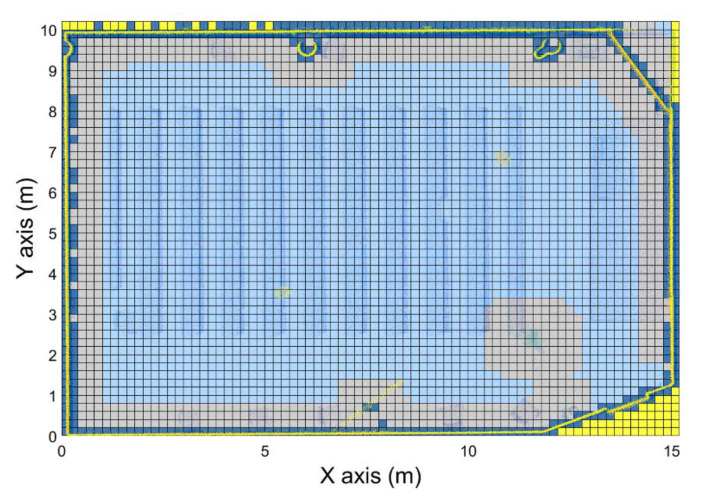
Horizontal plane of voxels. Light blue: Empty voxels. Dark blue: Occupied voxels. Grey: Security-offset voxels. Yellow: Exterior voxels.

Finally, all the empty voxels near an occupied one are classified as a “Security-offset” to avoid possible collisions during the navigation. Accordingly, a minimum-security distance is defined depending on the UAV dimensions and must be large enough to ensure the security of the system during navigation. The number of voxels defined as a security offset around an occupied one is calculated using Equation (3). An exception to this “Security-offset” is considered for doors: If a door area is labeled as “Security-offset,” their voxels are re-classified to “Empty” to ensure navigation between rooms. This discretization is an input for the algorithm that generates the navigation map (Figure 10).

(3) $$N=\;\left(ceil\left(\frac{security\;distance}{voxel\;size}\right)\times 2\right)+1$$

#### 2.1.3. Navigation Map Generation 

After point cloud voxel classification, a navigation map is created for each room separately, the doors being the connection component between rooms. Door locations are obtained as described in Section 2.1.1, and act as the target positions for the navigation map generation because the UAV has to pass through the door to navigate from one room to another.

The navigation map consists of a matrix with the same dimensions as the voxelization where each navigable voxel is given a value according to the following rules:

- Target voxel is the empty voxel that contains the door position and labeled with a “1”.
- Navigable voxels are empty voxels directly connected to the target voxel, meaning that there is at least one path from the voxel to the target. The label of these voxels depends on the number of surrounding voxels and the distance to the target.
- Nonnavigable voxels are empty voxels that are not directly connected to the target voxel, meaning that there is no possible path between them and the target voxel. These voxels are given a label value of “−1”.

**Figure 11:**
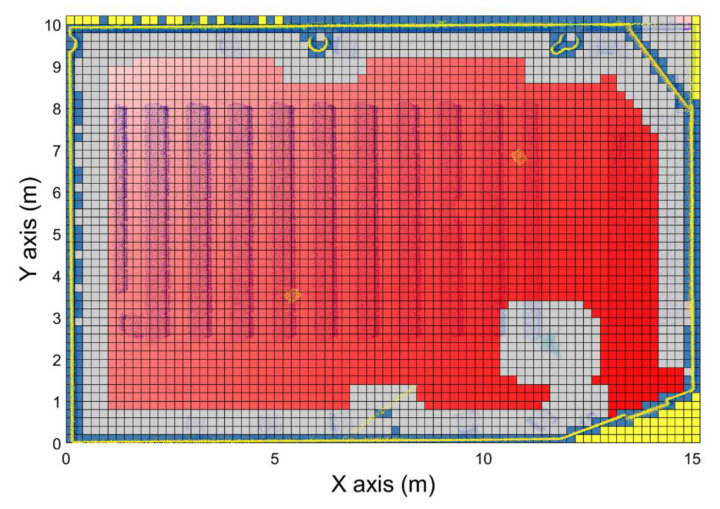
Horizontal plane of voxels of the navigation map (red: Navigable voxels, the transparency shows the value of each voxel; grey: Security offset; yellow: Exterior voxel; dark blue: Occupied voxel).

**Figure 12:**
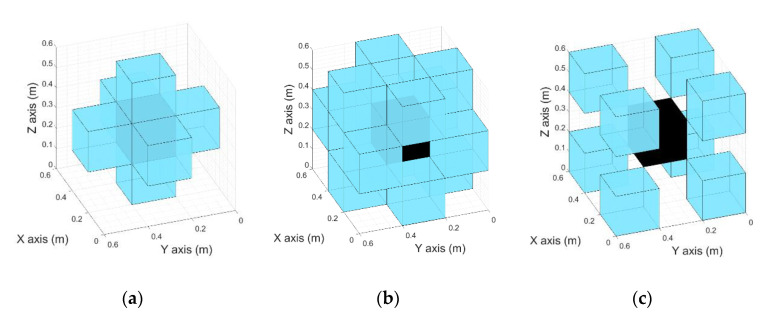
Different relations between voxels (black: Study voxel. Blue: Surrounding voxels). (**a**) Common face. (**b**) Common edge. (**c**) Common vertex.

The navigation map is generated just considering the target position. In this way, this navigation map (Figure 11) can be used to calculate the path between each navigable position of the room and the target. This path can also be navigated in both ways, selecting the target voxel as the initial or the final position. The label for the navigable voxels depends on the adjacency relationship to their surrounding 26 voxels in the next manner (Figure 12):

- Common face: Voxels surrounding the study voxels that have a common face. This means that the surrounding voxels and the study one have two coincident indices.
- Common edge: Voxels surrounding the study voxel that have a common edge. This means that the surrounding voxels and the study one have one coincident index.
- Common vertex: Voxels surrounding the study voxel that have a common vertex. This means that the surrounding voxels and the study voxel do not have any coincident indices.

**Figure 13:**
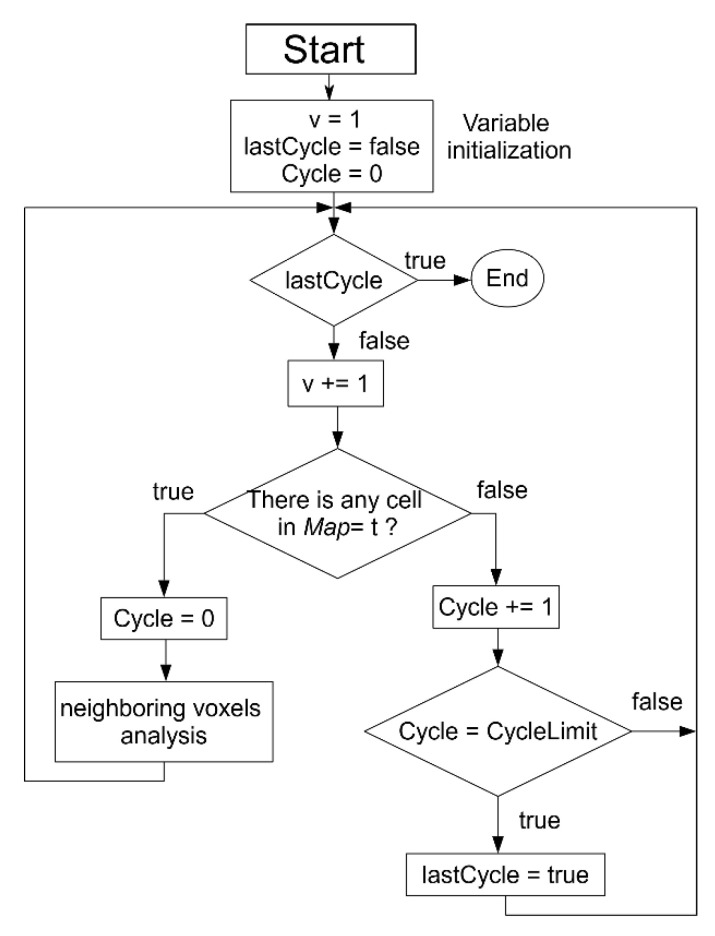
Navigation map generation workflow.

Figure 13 shows the workflow of the navigation map generation (Figure 11). First, an initial value of “−1” (nonnavigable) is given to all the voxels. Then, a value of “1” is given to the target position, the starting point for the navigation map generation. Surrounding cells are later labeled with a weighted sum of their adjacency: Common face, adds “1,” Common edge, adds 2, and Common vertex adds 3. If the result is lower than the previous label, this new value is assigned to the voxel. Thus, each voxel of the navigation map has the lowest possible weight. The maximum weight corresponding to the Common vertex with a “3” value is used as stop criteria variable *CycleLimit.* This variable is used to detect when the navigation map is completely calculated, i.e., labeling all the navigable voxels. When the system has not found any next study voxel in 3 loops, this means that there are no more navigable voxels to be studied, so the navigation map is already completed. A variable v is used to set-up the voxels to be analyzed for each loop iteration, starting with “1” that means that only the target voxel is a candidate and the 26-voxel vicinity is analyzed and updated. For iteration v = 2, every voxel of the navigation map labeled with “2” is analyzed and their vicinity updated, incrementing the *v* index until the generation map is completed. 

As aforesaid, one of the main objectives of this development is to speed up the path planning algorithm to support near-real-time path calculation. Thus, the presented approach consists of pre-processing as much of the computational costly procedures as possible. 

Rooms could contain more than one door, acting as connecting spaces between two adjacent rooms. For these connecting rooms, we create two kind of maps: Navigation and linking maps. A navigation map is calculated per door, each one being considered as the target position for the correspondent map generation. In addition, a number of linking paths connecting a pair of doors is calculated for every combination of door pairs. With this method, to obtain a path from one room to another one that does not share any common doors with the origin, we only need to query the linking path already calculated in this pre-processing step. Accordingly, path planning consists of calculating the path from the initial position to the target door in the initial room and the path from the target door in the destination room to the final position, making use of the predefined linking paths between the rooms. 

To end the pre-processing methods, a navigation graph is derived based on the map obtained in the previous step. The navigation graph is an undirected structure, where each node is a room and each link is a door, containing all the information about the connection between rooms. The graph supports the calculation of the rooms the vehicle has to pass through to navigate to the goal location. The initial node for graph generation is the room with the largest number of doors, populating the connections to the next rooms to obtain the navigation map for the entire point cloud.

**Figure 14:**
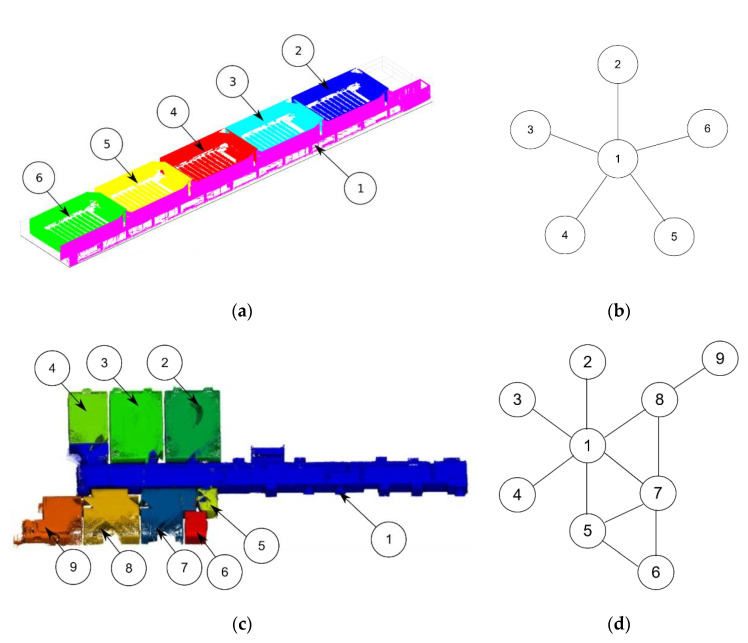
Navigation graph generation. (**a**) Numeration of rooms of the first example. (**b**) Navigation graph generated of the first example. (**c**) Numeration of rooms of the second example. (**d**) Navigation graph generated of the second example.

Figure 14 shows the navigation map generated for the examples used, where the first example is composed by five rooms connected to one corridor. The corridor is the room with the largest number of doors, and so is used as the initial node for the graph generation. This is the simplest graph possible because all the rooms are connected only to a corridor space and there do not exist any loops in the graph. The second example yields a more complex distribution including three different loops between rooms numbered 1-5-7, 5-6-7, and 1-7-8. 

The navigation map is used to calculate the rooms that the vehicle may pass through to go from one room to another. This is done with an iterative algorithm that explores all the possible routes fulfilling the following requirements:

- The route cannot pass through the same room twice.
- If two or more routes pass through the same room, the one containing the lower number of rooms is selected as the best route.
- If two routes are valid and do not share any part of the path, that containing the lowest number of waypoints, i.e., the shortest path, is selected.

In the case of navigation maps with loops, as in the second example, the second rule solves the problems of multiple possible routes. For example, if the vehicle must go from room 1 to room 9, several routes that fulfil the first rule are possible: 1-8-9, 1-7-8-9, 1-5-7-8-9, and 1-5-6-7-8-9. All the candidate routes share the last rooms of the paths, being the first candidate selected as the best route. In other cases, more than one path that fulfil the first and second rules are possible. If the vehicle must go from room 1 to room 6, two routes fulfil the first and second rules: 1-5-6 and 1-7-6, the candidate route selected being 1-5-6 because it has a lower number of waypoints. This path calculation algorithm is explained in depth in the next section.

### 2.2. Path Planning

The navigation map generated in the last step is used by the path planning algorithm to calculate the route that the vehicle must follow to go from one point to another. The first step to calculate the path between two points is to locate the corresponding rooms for the initial and final positions. This is achieved using the room area calculated during point cloud segmentation in Section 2.1.1. If the initial position and the final position are in the same room, the navigation map of the room has to be updated, because the target position of the previous navigation map was the door. If the initial and final positions are not in the same room, the rules explained in the previous section are used to calculate the best route. 

In both cases, the path planning algorithm starts at the initial position. This position has to be at a navigable voxel; in other cases, this position is not valid. If it is at the floor height, i.e., the vehicle is landed; the algorithm uses the firsts waypoints to take-off to a predefined height, 1 m in our case. The algorithm verifies that the voxels crossed in the take-off maneuver are navigable, otherwise marking the initial position as not valid. Then, the position after take-off is selected as the new initial position to calculate the path using an iterative algorithm from the initial voxel, studying the value of the 26 voxel vicinities around it and selecting the voxel with the lowest value as the next waypoint. Such a waypoint is treated as the initial point for the following loop, extending the watershed for the path until complete, occurring when the target position with a “1” label is reached. The waypoints of the path are centered in the voxel selected as the next study voxel and the number of waypoints of the route is accumulated to select the shortest path when needed, and according to the third rule for the route calculation algorithm explained in the previous section. Algorithm 2 shows how the path planner algorithm calculates the route using the navigation map created previously.
**Algorithm 2** Path Planning Algorithm**Require:** NavigationMap: NM, InitialPos: I.[Ix, Iy, Iz] = I;firstCycle = true;Route.InitializeRoute;**while** not(EndReached) **do**//Look for the next position in the 26 voxels around the current position      f**or** x = Ix − 1:Ix + 1            f**or** y = Iy − 1:Iy + 1                **for** z = Iz − 1:Iz + 1                  **if** (firstCycle) **then**                     firstCycle = false;                     minVal = NM (x, y, z);                     NextPos = (x, y, z);                        **else then**                          **if** NM (x, y, z) < minVal **then**                          NextPos = (x, y, z);                          minVal = NM (x, y, z);                         **endif**            **endif**                 **endfor**            **endfor**       **endfor**       *//Add the next position to the path and look if the target has been*
       *reached*       Route.AddPos(NextPos);       **if** (NM(NextPos) == 1) **then**EndReached = true;       **else then**            [Ix, Iy, Iz] = NextPos;            firstCycle = true;       **endif****endwhile**

**Figure 15:**
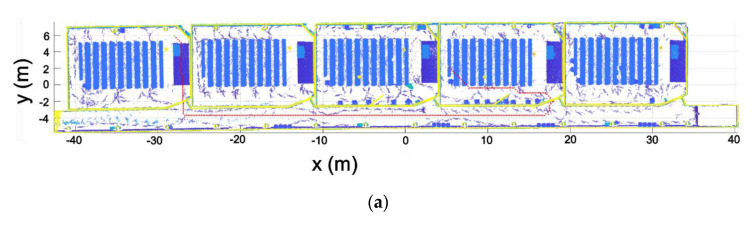

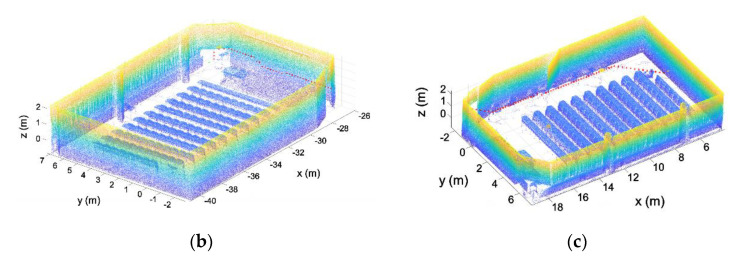
Path calculated between two different rooms (red: Waypoints; green: Initial position; yellow: Final position). (**a**) Complete path. (**b**) Initial room path. (**c**) Final room path.

This path planning algorithm has been designed to be implemented in UAVs for contact inspection purposes (Figure 15). According to this, the final position also has an orientation vector needed to perform the contact. Usually, this orientation is perpendicular to the wall/object surface in the contact point selected. A similar procedure to the location of the initial position after the take-off maneuver is implemented for the final position, but in this case, the target orientation vector is perpendicular to the wall and the offset distance was set to 1.5 m. As a result, the path is calculated for the UAV to navigate from the initial position to the final POSE (position and orientation) to enable the contact inspection. At this point, the contact-inspection system developed in previous works [11] takes control of the vehicle to drive the approach to the stable contact. 

**Figure 16:**
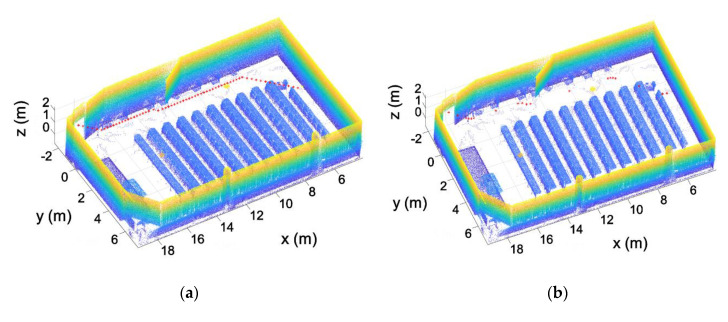

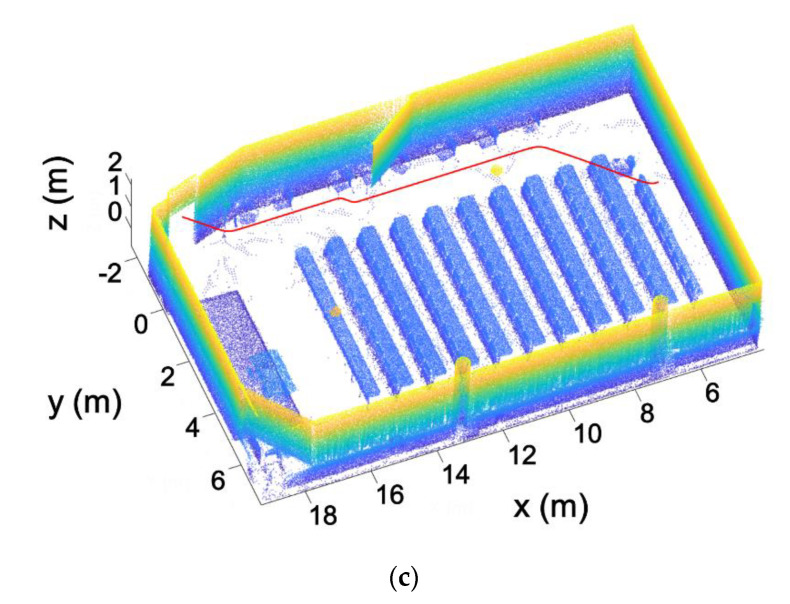
Path smoothing (red: Waypoints). (**a**) Initial path. (**b**) Path smoothed and simplified. (**c**) Final path after smoothing.

Once the path is calculated, a smoothing algorithm segments the path in the linear components of the route. The joint between each pair of consecutive lines of the route is replaced by a circumference tangent to both lines, with a defined radio $r_{c}$. As the route is defined by waypoints, the tangent arc is simplified by using a number of waypoints pre-defined by the user. This smoothing is applied only when both lines are longer than the radio $r_{c}$. Further, if a line is longer than a minimum length $l_{Min}$, it is simplified by three points at the beginning, at the middle, and at the end of the line, thus drastically reducing the total number of waypoints in the path. In the example shown in Figure 16, the number of waypoints of the path is reduced from 65 to 19 waypoints. This smoothing is done with a ratio $r_{c}=0.6\;m$, 4 waypoints to define the arc and a minimum length $l_{Min}$ to simplified lines of 0.6 m. 

**Figure 17:**
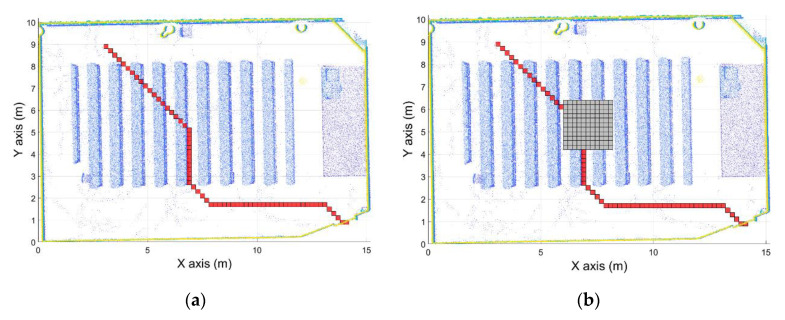
Obstacle detected in the path calculated. (**a**) Initial path calculated. (**b**) Obstacle detected with the security offset around the obstacle defined.

The point cloud used to calculate the navigation map was taken in a pre-processing step, meaning that new obstacles can be placed in the room. These obstacles, such as lockers placed after the first point cloud acquisition, can cause an accident if the path calculated goes through the object. Thus, when the UAV navigates using this route, the system is going to collide with the obstacle (Figure 17). Due to this, an obstacle avoidance path planner system has been developed. This system is able to calculate new routes to modify the previous route to avoid the obstacle. Thus, when the vehicle detects an obstacle when it is navigating through the calculated route, a new study area centered in the obstacle is defined. To create this area, the first step is to define the position of the obstacle and create a security offset around it, in order to define the area where the UAV cannot navigate to avoid collisions. The same security offset used to generate the navigation map is used. 

**Figure 18:**
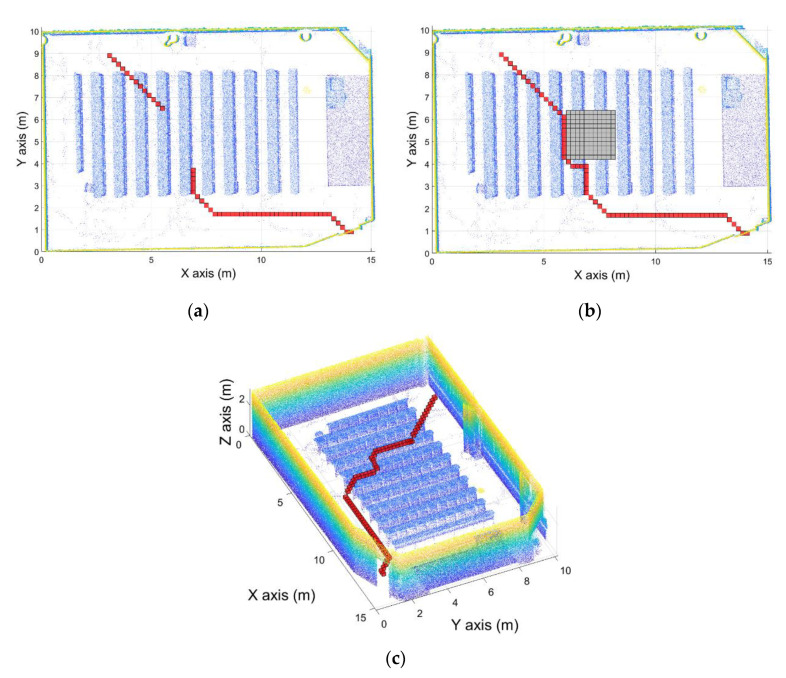
Obstacle avoidance path planner. (**a**) Path segmented before and after the obstacle. (**b**) New path calculated avoiding the obstacle with a security offset. (**c**) Path calculated.

Once the obstacle area is defined, the route is divided into two parts, before and after the obstacle (Figure 18a). Therefore, the objective now is to calculate the path that joins the two segments avoiding the obstacle. To do this, a new navigation map must be calculated considering the obstacle and the security offset around it. In order to make this operation as fast as possible, instead of calculating the navigation map in the entire room, a study area around the obstacle is defined. This area around the obstacle position is calculated using the security offset defined previously. Thus, the study area is defined as 2 times the security offset around the obstacle. The navigation map is calculated in this area using the same algorithm used in the pre-processing step, defined as the target of the initial position of the second segment, the segment after the obstacle. Then, the path planning algorithm is used in this study area using as the initial position the final point of the segment before the obstacle. In this way, a new route that avoids the obstacle has been calculated (Figure 18).

## 3. Result and Discussion

### 3.1. Case Study

**Figure 19:**
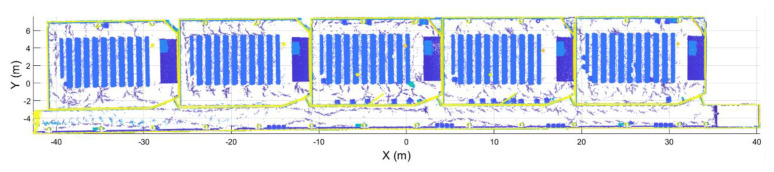
Case of study.

This study was carried out using a point cloud acquired at the Mining and Energy School at the University of Vigo (Figure 19). As can be seen in all the Figures used previously to explain the method, the point cloud is composed of six rooms: Five classrooms and a corridor that connects all of them. The size of the study area was approximately 1020 m^2^ (85 m × 12 m). The point cloud, which contains 4.5 million points, has been acquired with a ZEB-REVO Laser Scanner [33], that is, a LS with a maximum range of 30 m in optimal conditions, a field of view of 270° × 360°, and a scan range noise of ±30 mm. 

A voxel size of 20 cm has been used. The pre-processing step took 83.2186 s to execute using an Intel core i7 and 16 GB of RAM. The path calculation took around 0.0131 s to execute and calculate a new route. All the algorithms for the path planning method have been developed in MatLAB 2020b, using the Point Cloud Library.

### 3.2. Results 

**Figure 20:**
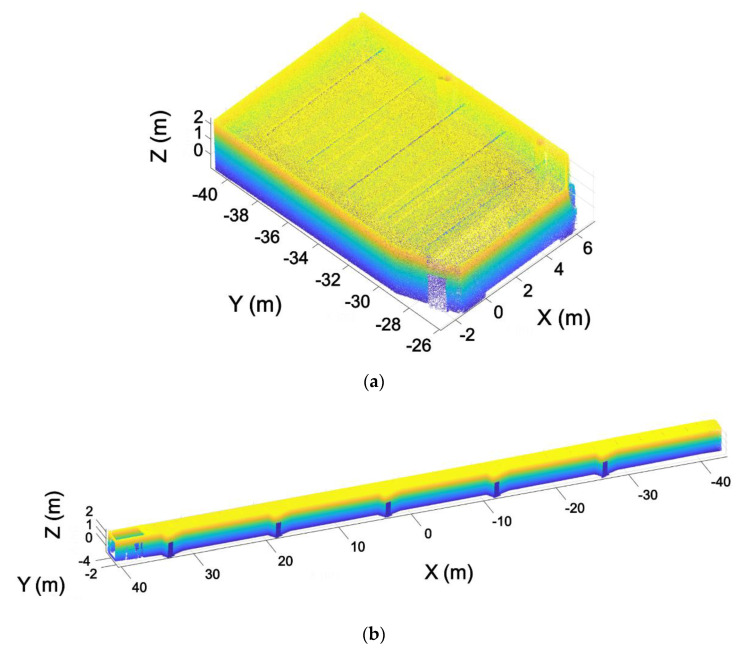
Segmentation results. (**a**) Standard room. (**b**) Corridor.

Regarding the segmentation process, Figure 20 shows the results of the segmentation of one of the rooms and the corridor point clouds. As can be seen, each point cloud is correctly segmented, containing only points that belong to the room. 

The method has been tested calculating multiple paths (Table 1). In all the cases, the algorithm has calculated a proper route that connects the initial position and the target one. The path planning algorithm developed is able to calculate the path between two points in an average time of 0.0079 s. These results demonstrate that the developed method can be used to calculate paths in real time. 

Regarding the obstacle avoidance path planner, the developed algorithm calculates a new path avoiding the obstacle in 0.05–0.06 s. For the case shown in Figure 18, the algorithms calculate the new path in 0.057265 s. 

**Figure 21:**
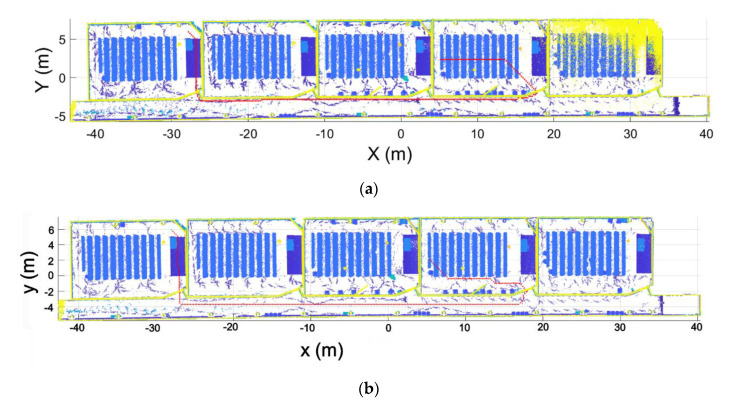
Routes (red dots) calculated between the same initial and final position. (**a**) Using an implementation of the A* path planning algorithm. (**b**) Using the modification of the A* path planning algorithm introduced in this manuscript.

If the path planning algorithm presented in this manuscript is compared with the previous A* implementation [34], an average speed up to 213 times higher is achieved. Table 2 shows the comparison of the execution times of both methods, the traditional A* and the method presented in this manuscript, comparing them. In both methods, a reliable path is calculated, as shown in Figure 21. 

In order to evaluate if the path planning algorithm is executed in real time, the time restrictions have to be defined. In this case, the velocity movement of the UAV is used to calculate this execution time restriction. Supposing that the UAV has a maximum speed of 1 m/s, that is a high velocity for indoor navigation. The average time used to calculate a path is 0.0079 s, meaning that the UAV could move 7.9 mm. In the case of the obstacle avoidance path planner, this algorithm takes an average of 0.05 s to been executed, so in this case with the maximum velocity of 1 m/s, the UAV could move 50 mm. Taking into account all this, the algorithm developed can be considered a real-time path planner. If the same time restrictions are applied to the A* algorithm implementation, with an average execution time of 1.51 s, the UAV could move 1.51 m at a velocity of 1 m/s. This means that the previous A* implementation cannot be considered a real-time path planner.

The execution time not only depends on the voxel size selected for the discretization or the point cloud size. The number of doors, the point cloud alignment, the interconnection between rooms, and other elements determine the execution time of the presented method. 

The algorithm presented in this work is a 3D path planner, which means it has been developed for 3D navigation of a UAV. It can be modified to been implemented in a 2D environment, adapting it to be used by other kinds of ROVs (remotely operated vehicles), like UGVs (unmanned ground vehicles) or autonomous surface vehicles.

## 4. Conclusions

This work presents a path planner algorithm developed for UAVs to navigate in indoor environments, only using as input the point cloud of the environment where the system is going to navigate. The main objective of this work is to calculate new paths in real time, which has been achieved, obtaining a speed up 213, comparing the new algorithm with the previous A* implementation. This algorithm is also orientated to perform contact inspection tasks autonomously. The method is divided into two parts. The first step has almost all the computational cost and the second part uses the data created by the first step to calculate new paths in real time. The algorithm developed has reached the following goals:

- The pre-processing step segments the entire point cloud into rooms, and each room is discretized, calculating the navigation map for each one using the doors as the target position.
- The algorithm calculates the navigation graph of the building where the system is going to navigate.
- The path planning algorithm uses the navigation graph to calculate the rooms that the UAV has to cross in order to go from an initial point in one room to a final point in another one.
- The path planner algorithm also uses the navigation map generated in the pre-processing step to calculate the path, making this calculation in real time.

To sum up, the developed algorithm fulfils all the requirements defined initially, where the most restrictive one is the execution time requirements. The developed path planning algorithm is executed in real time. 

In the future, this path planning algorithm is going to be implemented in the contact inspection system developed previously, also using a GPU and parallel programming strategies to optimize the algorithm. In this way, and autonomous contact inspection system based on UAVs is going to be developed. Moreover, the developed algorithm is going to be modified to make it a multivehicle path planning system, to perform contact inspection with different UAVs in an indoor environment at the same time.

## Figures and Tables

**Table 1:** Result of different path calculations.

N. Path	Initial Pos. (m)	Final Pos. (m)	Vec. Dir.	Time (s)	N. Waypoints.
1	[−27.9, 5.85, −0.8]	[4.2, 2.3, 1]	[−1, 0, 0]	0.008745	345
2	[−13.5, 5.85, −0.8]	[19.4, 2.3, 1]	[−1, 0, 0]	0.006259	340
3	[1.2, 5.85, −0.8]	[−10.6, 2.3, 1]	[−1, 0, 0]	0.00851	342
4	[17.2, 5.85, −0.8]	[−25.5, 2.3, 1]	[−1, 0, 0]	0.012885	263
5	[32, 5.85, −0.8]	[−40.6, 2.3, 1]	[−1, 0, 0]	0.006533	415
6	[−27.9, 5.85, −0.8]	[18.4, 2.5, 1]	[1, 0, 0]	0.008942	303
7	[−13.5, 5.85, −0.8]	[33.5, 3, 1]	[1, 0, 0]	0.005645	289
8	[17.2, 5.85, −0.8]	[3.4, 3, 1]	[1, 0, 0]	0.009818	151
9	[17.2, 5.85, −0.8]	[−11.5, 3, 1]	[1, 0, 0]	0.005481	239
10	[32, 5.85, −0.8]	[−26.7, 3, 1]	[1, 0, 0]	0.006902	377

**Table 2:** Comparation between the execution time of the A* path panner and the presented path planner.

N. Path	Time A* (s)	Time Modified A* (s)	$\mathrm{Speed}\;\mathrm{Up}\;\left(\frac{Time\;A*}{Time\;Modified\;A*}\right)$
1	1.550767	0.008745	177.33
2	1.576993	0.006259	251.96
3	1.416444	0.00851	166,44
4	1.538592	0.012885	119.41
5	1.591487	0.006533	243.61
6	1.411514	0.008942	250.05
7	1.522057	0.005645	269.63
8	1.339394	0.009818	136.42
9	1.509533	0.005481	275.41
10	1.647614	0.006902	238.72

## Data Availability

Data sharing not applicable.

## References

[1] Greenwood W.W., Lynch J.P., Zekkos D. (2019). Applications of UAVs in Civil Infrastructure. J. Infrastruct. Syst..

[2] Seo J., Duque L., Wacker J.P. (2018). Field Application of UAS-Based Bridge Inspection. Transp. Res. Rec. J. Transp. Res. Board.

[3] Ellenberg A., Kontsos A., Moon F., Bartoli I. (2016). Bridge related damage quantification using unmanned aerial vehicle imagery. Struct. Control. Health Monit..

[4] Remondino F., Barazzetti L., Nex F.C., Scaioni M., Sarazzi D. (2012). UAV Photogrammetry for Mapping and 3d Modeling–Current Status And Future Perspectives. ISPRS Int. Arch. Photogramm. Remote. Sens. Spat. Inf. Sci..

[5] Teng G.E., Zhou M., Li C., Wu H.H., Li W., Meng F.R., Zhou C.C., Ma L. (2017). Mini-UAV Lidar for Power Line Inspection. ISPRS Int. Arch. Photogramm. Remote. Sens. Spat. Inf. Sci..

[6] Márquez F.P.G., Ramírez I.S., Segovia I. (2019). Condition monitoring system for solar power plants with radiometric and thermographic sensors embedded in unmanned aerial vehicles. Meas. J. Int. Meas. Confed..

[7] Sony S., LaVenture S., Sadhu A. (2019). A literature review of next-generation smart sensing technology in structural health monitoring. Struct. Control. Health Monit..

[8] Ikeda T., Yasui S., Fujihara M., Ohara K., Ashizawa S., Ichikawa A., Okino A., Oomichi T., Fukuda T. Wall contact by octo-rotor UAV with one DoF manipulator for bridge inspection. Proceedings of the 2017 IEEE/RSJ International Conference on Intelligent Robots and Systems (IROS).

[9] Sanchez-Cuevas P.J., Ramon-Soria P., Arrue B., Ollero A., Heredia G. (2019). Robotic System for Inspection by Contact of Bridge Beams Using UAVs. Sensors.

[10] Han H., Wang J., Wang J., Tan X. (2015). Performance Analysis on Carrier Phase-Based Tightly-Coupled GPS/BDS/INS Integration in GNSS Degraded and Denied Environments. Sensors.

[11] González-Desantos L., Martínez-Sánchez J., González-Jorge H., Navarro-Medina F., Arias P. (2020). UAV payload with collision mitigation for contact inspection. Autom. Constr..

[12] Potorti F., Palumbo F., Crivello A. (2020). Sensors and Sensing Technologies for Indoor Positioning and Indoor Navigation. Sensors.

[13] Deng H., Fu Q., Quan Q., Yang K., Cai K.-Y. (2020). Indoor Multi-Camera-Based Testbed for 3-D Tracking and Control of UAVs. IEEE Trans. Instrum. Meas..

[14] Xin C., Wu G., Zhang C., Chen K., Wang J., Wang X. Research on Indoor Navigation System of UAV Based on LIDAR. Proceedings of the 2020 12th International Conference on Measuring Technology and Mechatronics Automation (ICMTMA).

[15] Chen Y., Gonzalez-Prelcic N., Heath R.W. Collision-Free UAV Navigation with a Monocular Camera Using Deep Reinforcement Learning. Proceedings of the 2020 IEEE 30th International Workshop on Machine Learning for Signal Processing (MLSP).

[16] Moussa M., Zahran S., Mostafa M., Moussa A., El-Sheimy N., Elhabiby M. (2020). Optical and Mass Flow Sensors for Aiding Vehicle Navigation in GNSS Denied Environment. Sensors.

[17] Youn W., Ko H., Choi H.S., Choi I.H., Baek J.-H., Myung H. (2020). Collision-free Autonomous Navigation of a Small UAV Using Low-cost Sensors in GPS-denied Environments. Int. J. Control Autom. Syst..

[18] Aggarwal S., Kumar N. (2020). Path planning techniques for unmanned aerial vehicles: A review, solutions, and challenges. Comput. Commun..

[19] Dijkstra E.W. (1959). A note on two problems in connection with graphs. Numer. Math..

[20] Yang L., Qi J., Xiao J., Yong X. A literature review of UAV 3D path planning. In Proceeding of the 11th World Congress on Intelligent Control and Automation.

[21] Iacono M., Sgorbissa A. (2018). Path following and obstacle avoidance for an autonomous UAV using a depth camera. Robot. Auton. Syst..

[22] Valenti F., Giaquinto D., Musto L., Zinelli A., Bertozzi M., Broggi A. Enabling Computer Vision-Based Autonomous Nav-igation for Unmanned Aerial Vehicles in Cluttered GPS-Denied Environments. Proceedings of the 2018 21st International Conference on Intelligent Transportation Systems (ITSC).

[23] Lakas A., Belkhouche B., Benkraouda O., Shuaib A., Alasmawi H.J. A Framework for a Cooperative UAV-UGV System for Path Discovery and Planning. Proceedings of the 2018 International Conference on Innovations in Information Technology (IIT).

[24] Yan W., Culp C., Graf R. (2011). Integrating BIM and gaming for real-time interactive architectural visualization. Autom. Constr..

[25] Peralta F., Arzamendia M., Gregor D., Reina D., Toral S. (2020). A Comparison of Local Path Planning Techniques of Autonomous Surface Vehicles for Monitoring Applications: The Ypacarai Lake Case-study. Sensors.

[26] Korkmaz M., Durdu A. Comparison of optimal path planning algorithms. Proceedings of the 2018 14th International Conference on Advanced Trends in Radioelecrtronics, Telecommunications and Computer Engineering (TCSET).

[27] Huang X., Cao Q., Zhu X. (2019). Mixed path planning for multi-robots in structured hospital environment. J. Eng..

[28] Maoudj A., Hentout A. (2020). Optimal path planning approach based on Q-learning algorithm for mobile robots. Appl. Soft Comput..

[29] Hernández-Mejía C., Vázquez-Leal H., Sánchez-González A., Corona-Avelizapa Á. (2019). A Novel and Reduced CPU Time Modeling and Simulation Methodology for Path Planning Based on Resistive Grids. Arab. J. Sci. Eng..

[30] Ambroziak T., Malesa A., Kostrzewski M. (2018). Analysis of multicriteria transportation problem connected to minimization of means of transport number applied in a selected example. WUT J. Transp. Eng..

[31] Dang T., Tranzatto M., Khattak S., Mascarich F., Alexis K., Hutter M. (2020). Graph-based subterranean exploration path planning using aerial and legged robots. J. Field Robot..

[32] Li H., Savkin A.V., Vucetic B. (2020). Autonomous Area Exploration and Mapping in Underground Mine Environments by Unmanned Aerial Vehicles. Robotica.

[33] Ltd G. ZEB-REVO Laser Scanner. https://download.geoslam.com/docs/zeb-revo/ZEB-REVOUserGuideV3.0.0.pdf.

[34] González-Desantos L.M., Martínez-Sánchez J., González-Jorge H., Arias P. (2020). Path planning for indoor contact inspection tasks with UAVS. Int. Arch. Photogramm. Remote Sens. Spat. Inf. Sci..

[35] Díaz-Vilariño L., Verbree E., Zlatanova S., Diakite A. (2017). Indoor modelling from SLAM-based laser scanner: Door detection to envelope reconstruction. Int. Arch. Photogramm. Remote Sens. Spat. Inf. Sci..

[36] Fischler M.A., Bolles R.C. (1981). Random sample consensus: A paradigm for model fitting with applications to image analysis and automated cartography. Commun. ACM.

[37] González-Desantos L.M., Díaz-Vilariño L., Balado J., Martínez-Sánchez J., González-Jorge H., Sánchez-Rodríguez A. (2018). Autonomous Point Cloud Acquisition of Unknown Indoor Scenes. ISPRS Int. J. Geo-Inf..

